# Semi-Monthly Meeting Rochester Medical Society

**Published:** 1874-12

**Authors:** J. O. Roe

**Affiliations:** Secretary


ART. III.—*Semi-Monthly Meeting of the Rochester Medical Society* Reported by J. O. ROE, M. D., Secretary.

Dr. WM. S. ELY, President in the chair.

After preliminary business Dr. Stoddard then presented a specimen with the following history:

Mrs. E., aet. 27 years, admitted to Rochester City Hospital, Aug. 10th, 1874.

Patient first menstruated at age of 13 years, always regular, flow normal in quantity and painful during first three days, at intervals troubled with leucorrhœa. The patient feels quite certain that the full period of gestation was reached July 12th,—last menstruation occurring about April 15th, 1873.

About four weeks since, July 18th, pain occurred resembling labor pains. A physician—(Homœopath)—was called, who after attendance for a day quieted the patient with morphia. This condition occurred several times, three other Homœopathic physicians attended her at intervals.

August 8th. Seen by Drs. Whitbeck, Dean, Little and Montgomery. Appearances externally that of pregnancy at full term. At this examination no os uteri could satisfactorily be recognized, what was supposed to be the occluded os lay high up behind the symphysis pubis. All efforts to enter the cervical canal were failures. The child was dead and easily distinguished. The head presented in the right iliac region.

August 10th. In consultation it was decided to operate by incision as near the supposed os as possible and to remove the child by version.

This was done at 3:15 P. M., under ether, Dr. J. F. Whitbeck operating. Present, Drs. F. H. Montgomery, Langworthy, Little, W. S. Ely and Stoddard.

An incision was made through the tissue covering the presenting portion of the head of the child. The left hand passed and the feet found in the left side; the child was drawn down and delivered, the whole period occupying about thirteen minutes—some hemorrhage followed. It was decided to remove the placenta at once, which was done with great difficulty, it being adherent and requiring considerable force to separate it. The cord dark and discolored was about two feet long.

Severe hemorrhage followed which was controlled by ice and injections of Sol. Per. Sulph. Iron, (but not until very severe hemor rhage had taken place.) Patient became extremely exhausted at 4-15, pulse 160, respiration 52.

Stimulants were freely given—patient gradually sank and died at 6:15.

*Post Mortem*—August 11th, 10 A. M. On opening abdominal cavity a large quantity of coagula and blood found. The uterus was high up behind and attached to the bladder. From the left ovary started a sac extending backward and downward, filling the cavity of the pelvis, and attached to the posterior surface of the uterus. This contained a large quantity of clots. The site of the placenta was over the surface of the ovary and beyond.

The uterus was enlarged.

| | |
|---|---|
| Length of cervical canal, . . . . . | $3\frac{1}{2}$ in. |
| Length of uterine cavity, . . . . . | $3\frac{1}{2}$ " |
| Whole length, . . . . . . . . | 7 " |
| Thickness of body of uterus, . . . . | $1\frac{1}{2}$ " |
| Width of fundus, . . . . . . | $3\frac{3}{4}$ " |
| Thickness of wall, . . . . . . | $\frac{5}{16}$ " |

CHILD.

| | |
|---|---|
| Weight, . . . . . . . | 8 pounds. |
| Length, . . . . . . . | 20 inches. |
| Circumference of head, . . . . | 15 " |
| Circumference of chest, . . . . | $13\frac{1}{2}$ " |

*Discussion*—Dr. Montgomery questioned whether it would not have been giving the woman a better chance had the doctor stopped after removal of the fœtus, and not attempted to remove the placenta.

Dr. Little concurred with Dr. Montgomery, stating that the woman got on nicely until the placenta was removed, when hemorrhage began, and the woman sank rapidly. He thought, had the placenta been left in and allowed to be expelled as a foreign substance it would have given the woman a chance.

Dr. Whitbeck said, he would have done so had he supposed or known, such a condition of things existed, but he thought he was removing the placenta from the cavity of the uterus.

At the request of Dr. Little, Dr. Hovey reported the following case. Mrs. G. was taken in labor at full term, primipara. At the end of twenty-four hours he saw the patient and after a careful examination it was ascertained that there was an occlusion of the os tincæ. It was decided to make an incision through the uterus for the delivery of the child. This was done at the pending part. The incision was two inches in length. Labor pains continued, and the opening dilated with the contractions of the uterus, and at the end of two and a half hours a healthy female child was born, weighing nine and a half pounds. Patient recovered without one bad symptom.

Dr. Dean then opened the discussion on Puerperal Fever—as follows:

Résumé—Puerperal Diseases and Puerperal Fever.

Dr. Dean referred to only such of the puerperal diseases as have come most frequently under his own observation. He said he should have spoken much more confidently of the nature and treatment of puerperal diseases, especially puerperal fever, several years ago than now.

Obstetrical literature of our day abounds in excellent essays and carefully made clinical observations in this department of medicine.

Teachers and practitioners of midwifery who taught the medical mind in this department twenty-five to fifty years ago, were very comprehensive in their nosology, comprising all the local inflammations of the puerperal patients under the general name of puerperal or child-bed fever; thus including under one head—diseases in the vaginal connective tissue, the uterus, fallopian tubes, ovaries, pelvic peritoneum, crural vein, &c., &c.

Before any discussion of the subject it seems proper to allude to the blood condition of pregnancy. Soon after impregnation the quantitative relation of the blood corpuscles undergoes a material change; the white corpuscles became relatively largely increased, and many of our best pathologists declare that the increase of the white corpuscles is *absolute;* the red corpuscles are relatively very much diminished, but it is not certain that the red corpuscles are *less* than in the unimpregnated state. This altered condition of the blood increases its coagulability, a condition technically demonstrated *hyperinosis*—a condition of "purulence," rather a condition strongly predisposing to purulence.

This blood change recognized it would seem that we are better prepared to anticipate and to appreciate many of the maladies of the puerperal state; especially the suppurative inflammations in perimetric tissues, the mammary gland, etc., etc., as also many of the fœtal and infantile diseases, the hepatic, duodenal and peritoneal diseases found in still born children; the jaundiced and other diseased conditions, found in early infancy.

For convenience of discussion, apart from essential puerperal fever—püerperal diseases may be arranged under the heads of

*traumatic*, and what are generally denominated *auto-genetic* diseases, in contra-distinction to heterogenetic or those originating from causes external to the patient.

According to the doctor's own observation the most common malady of the parturient patient is some form or degree of cellulitis; it may be no more than a limited inflammation of the submucous connective tissue of the vagina, traumatic in its origin, from detention of the fœtal head perhaps or the pressure of instruments, &c.

The next most frequent malady in the puerperal state, within the doctor's experience is some form or degree of metritis, or some of the forms of peritonitis, consequent generally upon endometritis. In primiparous births it is perhaps rare that patients do not sustain a considerable endometritic lesion of the cervix; and not unfrequently the cervical parenchyma is so much injured that inflammation will extend externally from the uterine cervix up into the perimetric tissues, producing all the consequences of endometritis, which is the more pathological lesion.

According to the estimate of the best living and recently deceased obstetricians and gynæcologists; this progress of puerperal perimetric disease—whether within or without the peritoneal cavity, is from the uterine cervix through the uterine cavity and fallopian tubes terminating in abscess or otherwise.

Puerperal peritonitis is considered by most writers as the most frequent of the puerperal diseases; may be primary or secondary, general or partial. If primary, appears in from one to three days after child birth, and may be independent of any of the lesions of the pelvic organs recognizable *post mortem*.

In a great majority of cases puerperal peritonitis is secondary to inflammation originating in some of the pelvic organs or tissues, as of uterus, fallopian tubes, of the ovaries or their enveloping tissues, and the disease appears later, after considerable progress, or even after the culmination of the more strictly local disease, and may occur during any period of the puerperal state; it may become general or remain as a partial peritonitis.

Primary puerperal peritonitis undoubtedly often originates from the general blood condition of pregnancy, leaving no trace of disease in pelvic organs beyond the peritoneal membrane.

Secondary puerperal peritonitis may also become general, indeed more often does, than lesions limited to the pelvic peritoneum.

Phlegmasia Dolens, milk-leg and crural phlebitis, are used by most authors as convertible terms. Obstetrical literature is plethoric with essays upon this subject, or, as the case may be, these subjects; and these contributions are from men of the best minds represented in this department of medicine.

The doctor said he should not attempt to differentiate the symptoms supposed to distinguish the one from the other disease. Previously he had regarded them as one and the same disease.

Prof. Fordyce Barker, of New York, of whose work on Puerperal Diseases, Dr. Dean said he was proud, as being in his (Dr. D's) estimation the best treatise in the English language upon kindred subjects, asserts the plurality of the diseases mentioned, and philosophically maintains his position. Thrombosis is allowed to be associated with both diseases, but claimed to be the *primary* condition in phlegmasia dolens, and the *phlebitis* the consequence; while crural *phlebitis* results from a poisoned condition of the blood of the patient without primary mechanical obstruction as in the former diseases. This is Dr. Barker's position as he (Dr. D.) understands it; as opposed to the opinion of many typical men who are equally emphatic in their expression of a contrary opinion; but the doctor said he was well satisfied with Dr. Barker's logic.

Pyæmia, septicæmia, infection and contagion as referring to puerperal disease are very differently appreciated by our most honored obstetricians and gynæcologists. Bearing upon this subject, Dr. Dean said he should consider himself culpable if he were to assume the care of a woman in labor while yet having, or having recently had the care of cases of puerperal fever or phlegmonous erysipelas. After eliminating the several features of puerperal disease, there yet ren ains the most to be dreaded accident or condition of the puerperal state; a condition unmarked by any of the symptoms so prominent in the traumatic anto-genetic diseases already referred to. The patient may be free from pain, and of every other symptom appreciable by herself as indicative of evil, while yet she may be in eminent danger; in a word, she is the subject of the poison of *essential puerperal fever*. She may or may not show abdominal or pelvic tenderness, certainly no more than in the most benign cases of child birth, neither will there necessarily appear tympanitic condition of abdomen. As a rule there will be found very slight lacteal secretion, also a scanty and fetid lochial discharge; but there will be found from the first an accelerated pulse and high temperature. The only point he said he had purposed to make from the subjected he had considered, was that of the *temperature of puerperal* fever patients, which, under limited qualifications he considered almost pathognomonic.

In benign puerperal cases we expect to find at some time during the first 72 hours some accelaration of pulse, some increase of temperature, some general and some local special tenderness, all of which generally subsides after quiet sleep, emptying of the bladder and the establishment of lactation. Some patients of a highly nervous temperament from repeated and severe after pains will exhibit quickened pulse, great tenderness and distension of the abdomen, but will not exhibit a protracted high temperature. If after the third day, with a quickened pulse, temperature above 102°, whether suffering from local tenderness or not, the doctor said he should predict prospective trouble, either a local purulence tending to abscess, or a non-malignant puerperal fever. In this estimate the doctor did not include any form of phlebites as apt to occur at a later puerperal period. Any local inflammation tending to abscess is comparatively benign and will be indicated by local symptoms. In absence of the several and more definitely marked symptoms of local inflammation, a temperature above 101° to 105°, after the third day, would be to his mind a very certain evidence of the existence of the malignant elements of *essential puerperal fever*.

A brief discussion followed.

Dr. Mallory said he had attended the post mortem examination of six cases of puerperal fever in Bellevue Hospital, and all contained pus in the peritoneum, and one in the uterus.

Dr. Starr enquired of Dr. Dean his treatment.

Dr. Dean said he had purposely omitted the treatment, but the therapeutics of these cases he could state in a very few words. They consist of quinia, opium and moderate catharsis. He also spoke of Dr. Barker's treatment with veratrum veride.

Dr. J. F. Whitbeck said he had met with an epidemic of this disease, and the fatal cases terminated usually in 84 hours.

Dr. Stoddard said he considered the symptom of high temperature of great significance in the disease.

Dr. W. S. Ely said he had limited experience with this disease. He had only had two case and both died. One of them died in 48 hours from commencement of attack but had no chill, tenderness, or distension of bowels. The other had chills, tenderness and distension of bowels; sank rapidly and died.

Dr. W. S. Ely next read a brief article on the determination of the age of the human embryo, and showed a specimen. A woman, 26 years old, who was nursing a 14 month's child, and had menstruated regularly for 6 months, commenced to flow 5 days after regular menstrual period, and discharged the ovum presented. As she did not suspect pregnancy, the age of the ovum must be determined from its appearance. Authorities differ widely as to length, weight, discrimination of sex, and development of fingers and toes, of ovum of three and four months. To prove which, statements were produced, extracted from the works of Cazeaux, Todd, Bowman, Tanner, Leishman, Meadows, Casper, Gray and Carpenter.

This specimen weighs 10 dr., 10 gr. and is five inches long. Its mouth is open, tongue visible, nose well formed, fingers distinct, and nails beginning to appear. It is regarded as of the male type, and as having entered on the fourth month of development, and the conclusion is, that we cannot, from inspection alone, of three or four months ovum determine with certainty within two weeks of its age.

On motion, the Society adjourned.

